# Distraction by deviant sounds is modulated by the environmental context

**DOI:** 10.1038/s41598-022-25500-y

**Published:** 2022-12-12

**Authors:** Fabrice B. R. Parmentier, Laura Gallego, Antonia Micucci, Alicia Leiva, Pilar Andrés, Murray T. Maybery

**Affiliations:** 1 Department of Psychology and Research Institute of Health Sciences, University of the Balearic Islands, Ctra. De Valldemossa, Km 7.5, Palma de Mallorca, Balearic Islands Spain; 2 Department of Psychology, University of Bologna, Bologna, Italy; 3 Department of Psychology, Universitat de Vic-Universitat Central de Catalunya, Vic, Spain; 4 School of Psychological Science, University of Western Australia, Perth, Australia

**Keywords:** Psychology, Human behaviour

## Abstract

Evidence shows that participants performing a continuous visual categorization task respond slower following the presentation of a task-irrelevant sound deviating from an otherwise repetitive or predictable auditory context (deviant sound among standard sounds). Here, for the first time, we explored the role of the environmental context (instrumentalized as a task-irrelevant background picture) in this effect. In two experiments, participants categorized left/right arrows while ignoring irrelevant sounds and background pictures of forest and city scenes. While equiprobable across the task, sounds A and B were presented with probabilities of .882 and .118 in the forest context, respectively, and with the reversed probabilities in the city context. Hence, neither sound constituted a deviant sound at task-level, but each did within a specific context. In Experiment 1, where each environmental context (forest and city scene) consisted of a single picture each, participants were significantly slower in the visual task following the presentation of the sound that was unexpected within the current context (context-dependent distraction). Further analysis showed that the cognitive system reset its sensory predictions even for the first trial of a change in environmental context. In Experiment 2, the two contexts (forest and city) were implemented using sets of 32 pictures each, with the background picture changing on every trial. Here too, context-dependent deviance distraction was observed. However, participants took a trial to fully reset their sensory predictions upon a change in context. We conclude that irrelevant sounds are incidentally processed in association with the environmental context (even though these stimuli belong to different sensory modalities) and that sensory predictions are context-dependent.

## Introduction

In many situations, filtering out task-irrelevant stimuli to focus on a task at hand plays an important role in efficient functioning. Yet, blocking such stimuli entirely can be counterproductive. For example, the reader of this article, however motivated to focus their attention on reading and to ignore the sound of nearby conversations or office noise, would benefit from detecting the unexpected sound of the fire alarm and from adapting their actions accordingly. Of relevance, one remarkable feature of our cognitive system is its capacity to rapidly detect unexpected stimuli. Abundant research has evidenced the existence of neurological and cognitive mechanisms responding to stimuli violating sensory predictions and resulting in the involuntary capture of our attention^1–7^. While adaptive, such mechanisms present one downside: the interruption of ongoing cognitive activities yields a transient reduction in ongoing task performance which, when attention-capturing stimuli are truly irrelevant, can be construed as distraction.

One class of stimuli repeatedly shown to grab attention are sudden changes (oddball, novel, or deviant stimuli) in a sequence of otherwise repeated or predictable (standard) sounds^6^. Such sounds induce an orienting response marked by specific electrophysiological responses^1,5,8,9^ and behavioral distraction in the task at hand^10,11^. The behavioral impact of unexpected sounds has been abundantly documented in simple categorization tasks (e.g., judging the parity of visual digits) involving task-irrelevant sounds e.g.,^12,13^. In such tasks, unexpected sounds lengthen response times to target stimuli in ongoing tasks due to the transient inhibition of motor actions^7,14–18^ and the involuntary shift of attention to and from the unexpected sound^1,10,19^. While typically studied in simple forced-choice categorization tasks^5,11–13^, the effects of task-irrelevant sounds have been observed also in other tasks such as duration judgement, go-nogo, visual matching, serial recall, and gap detection tasks^9,20–30^. One key finding is that unexpected sounds distract because they violate the cognitive system’s predictions rather than because they are rare per se^31–35^.

The literature reviewed above fits with the general notion that stimuli conveying surprise capture attention and can yield behavioral distraction^13,36–38^. Manipulations eliminating the element of surprise have been shown to reduce or eliminate distraction^31,34,35,39,40^. The question at the heart of this paper relates to the conditions under which a task-irrelevant sound constitutes a surprising event. Deviant sounds are typically defined as stimuli that deviate from the context of an otherwise repetitive or predictable sequence of sounds. Here, we consider a different type of context: the environmental context, that is, “incidental information about the environment in which the focal information is processed”^41^. Our aim was to examine whether the distractive impact of a task-irrelevant sound may be determined by the extent to which that sound is surprising within a given environmental context. To use an analogy, let us imagine a person walking down a busy street in Madrid. That person would most likely not pay particular attention to the sound of people speaking Spanish in their immediate surroundings but would notice the sound of people speaking Japanese. In contrast, on a busy street in Tokyo, the first would now stand out and be noticed, while the second much less so.

While the role of environmental context has not been examined with respect to auditory distraction, there is abundant evidence of its powerful impact on memory functioning, and some limited evidence of its impact on visual attention. Numerous studies show that memory performance is superior when retrieval takes place in the same environmental context as during encoding, and the impact of interfering stimulus lists is diminished if these are learned in an environment distinct from that of the to-be-remembered items^42–45^. The first type of finding has been observed by measuring free recall performance as a function of whether encoding and retrieval occurred in the same or in different places^45^, such as on land or under water^46^, in a small room or a large garden^47^, or in different rooms^48^. These effects have also been reported using recognition tasks^49,50^, and in the field of eyewitness testimony^51–53^. They are interpreted as the reflection of the implicit association between the context and the target information during encoding, allowing the context to act as an automatic cue at retrieval.

Of interest, environmental context also appears capable of modulating attentional functioning. For example, studies showing that environmental context in the form of a task-irrelevant background picture (black and white forest vs city scene) can influence the type of search (feature-search vs singleton)^54^ or modulate the impact of distractors^55^. In Cosman and Vecera’s study^54^, participants performed a visual search task across an array of stimuli presented over a task-irrelevant background picture. In the training phase of the experiment, participants were instructed to search for a circle among heterogenous nontarget items (feature-search) or for a different shape among homogenous nontargets (singleton search). Of importance, trials from each condition included the presentation of a background picture (forest or city). Following training, a test phase was presented in which either type of search could be used by participants. The results showed that the search strategy used by participants was influenced by the task-irrelevant picture present in the background, such that the background elicited the type of search formerly paired with it during the training phase. This indicates that task stimuli are processed in relation to the visual context. Further evidence shows that such contextual information is also capable of reinstating the reward value of a stimulus, increasing its impact when presented as a distractor^55^. In Anderson’s^55^ study, participants practiced a visual search task embedding two possible target stimuli (red or green circle containing a line segment, the orientation of which participants were instructed to categorize). The search array was presented over a background picture consisting of a forest or a city scene. Monetary rewards were set up such that a correct response to one target (e.g., red circle) yielded a reward within one context (e.g., forest) but not in the other (e.g., city), while the opposite arrangement applied to the other target (e.g., green circle). Following training, participants completed a test phase in which they were now instructed to respond to the line segment contained within a unique shape (e.g., circle among diamonds, or diamond among circles), again presented over forest or city background pictures, and in which one of the distractors was presented in a color formerly associated with target stimuli (red or green). The key finding was that the red and green distractors produced significantly more distraction when presented over the background picture with which they had previously yielded a reward. These results demonstrate that stimuli are processed in relation to the background picture, even if task-irrelevant, and that the latter can modify the way in which task stimuli are appraised.

The present study sought to join the two lines of research described above (distraction by unexpected sounds and context-dependent effects on attention) and extend them to a cross-modal situation (visual context and auditory distractors) to establish for the first time whether behavioral distraction by unexpected sounds can be modulated by the environmental context. We report two experiments using a modified cross-modal oddball task in which participants categorized left and right visual symbols in the presence of task-irrelevant sounds (A and B) and a background picture (forest or city scene). In contrast with past work in which black and white pictures were used to minimize the possible interference with a visual search task^54,55^, we used color pictures to maximize perceptual distinctiveness and realism (our primary task does not involve color and so possible color-specific interference was not an issue in our study). The crucial methodological aspect of our task laid in the probabilistic relationship between irrelevant sounds and environmental context. While sounds A and B were presented with equal probabilities across the experiment (as were the two types of contexts), A was more likely than B in one context and the reverse was true in the other context.

The capture of attention by unexpected sound has typically been interpreted as the result of the mismatch between the incoming sound and a neural model of past auditory events^56–58^ or between the incoming sound and a sensory prediction computed from such events^4,39,59^. The latter interpretation fits well within the general view that one important and quintessential function of the cognitive system is the building of mental models to form predictions against which incoming information is assessed^60,61^. The key issue in the present study is whether auditory predictions are computed based purely on past task-irrelevant auditory stimuli or on a more integrative assessment of the sounds in relation to other task-irrelevant contextual elements such as the visual background. The latter would suggest that sensory predictions are unlikely to be underpinned solely by posterior sensory brain regions and might involve, instead, more frontal regions participating in the integration of information at higher levels of the cognitive architecture. Such a proposition is line with findings suggesting that the within-modality processing of information mostly mobilizes primary sensory cortices while cross-modal information involves more extensive frontal networks^62^, and fits well with the hierarchical organization of the brain^63–65^.

**Figure 1:**
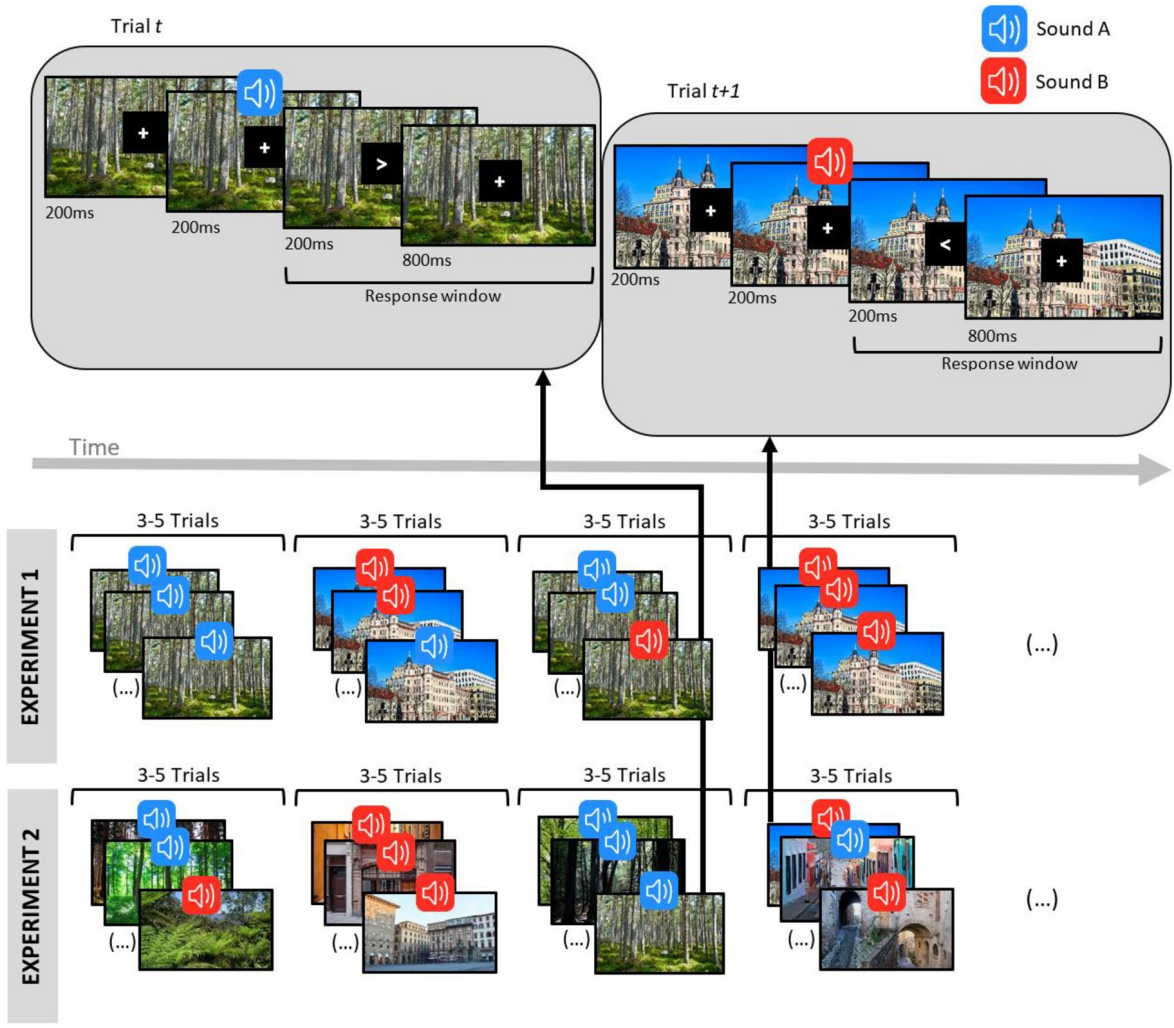
Schematic illustration of the task. The bottom part of the figure illustrates a sequence of runs sharing the same context (forest or city). The upper part illustrates the timeline of specific individual trials marked by black arrows). In each trial, a task-irrelevant background picture (environmental context consisting of the picture of a forest or a city scene) appeared and remained visible throughout the trial. This was followed by the presentation of a task-irrelevant sound (A or B), then by the appearance of the target stimuli (< or >), which participants categorized as pointing left or right. Sounds A and B, equiprobable across the task, consisted of 440 Hz and 1047 Hz sinewave tones (the allocation of these sound files to the sound conditions A and B was counterbalanced across participants). Within the forest context, sounds A and B were presented with probabilities of 0.882 and 0.118 respectively, while the reverse probabilities were used in the city context. Runs of 3 to 5 trials of the same context (forest or city) were presented in sequence (note that to avoid visual overcrowding of this figure, we display 3 pictures per run). In Experiment 1, these contexts consisted of a single picture each (randomly selected for each participant). In Experiment 2, the background picture changed on every trial, cycling through two sets of 32 randomly ordered pictures for each context (forest and city).

In this study, we carried out experiments in which participants categorized left/right symbols (“ < “ and “ > “) while instructed to ignore a task-irrelevant background picture and a task-irrelevant sound presented immediately before each visual target (Fig. 1). The background pictures depicted a forest or a city scene. Two sounds (A & B) were used equiprobably across the task but with different conditional probabilities within each of these two visual contexts: in the forest context, sounds A and B were presented with probabilities of 0.882 and 0.118 respectively, while in the city context, the opposite pattern was used. Under the context-independent hypothesis, task-irrelevant sounds should be processed relative to previous auditory events, such that only sounds construed as deviating from an otherwise predictable auditory stream should capture attention and lengthen response times in the primary visual task. In our experimental design, because sounds A and B are used with equal probabilities across the task, no sound should constitute a deviation and so response times should be similar for these two sounds irrespective of the environmental context. In contrast, according to the context-dependent hypothesis, task-irrelevant sounds should be appraised in relation to other elements of the task such as the environmental context, such that a sound should constitute a deviation whenever it violates sensory predictions within that context. If so, one sound (e.g., A) should elicit longer response times than the other (e.g., B) in the context in which A and B constitute deviant and standard sounds respectively.

## Analyses

In Experiments 1 and 2, we analyzed the mean proportion of correct responses and the mean response times (RTs) for correct responses. Effect sizes are reported as partial eta-square values for F tests, and as Cohen’s d_az_ for within-participant comparisons^66^. All t-tests were two-tailed, except in the comparison of distraction between Experiments 1 and 2, where we explicitly specify that a one-tailed test was used. In addition to frequentist statistics, we also report the Bayes Factor (BF_10_) to assess the credibility of the experimental hypothesis relative to that of the null hypothesis given the data. Values below 1/3 are considered as substantial to strong support for the null effect, while values above 3 are regarded as substantial to strong support for the presence of an effect^67,68^. Trials with response times faster than 200 ms were treated as anticipations and excluded from the analysis.

## Results

### Deviance distraction as a function of visual context

The mean proportion of correct responses (presented in Table 1) was analyzed using a 2 (Context: forest vs city) × 2 (Sound: A vs B) ANOVA for repeated measures. The main effect of Context was not significant, *F*(1,55) = 3.403, *MSE* = 3.9 × 10^–4^, *p* = 0.070, ${\eta }_{p}^{2}$ = 0.058, *BF*_*10*_ = 0.609, nor was that of Sound, *F*(1,55) = 0.061, *MSE* = 6.797 × 10^–4^, *p* = 0.806, ${\eta }_{p}^{2}$ = 0.001, *BF*_*10*_ = 0.148. The interaction between these factors was not significant either, *F*(1,55) = 1.373, *MSE* = 4.759 × 10^–4^, *p* = 0.246, ${\eta }_{p}^{2}$ = 0.024, *BF*_*10*_ = 0.342.

**Table 1:**
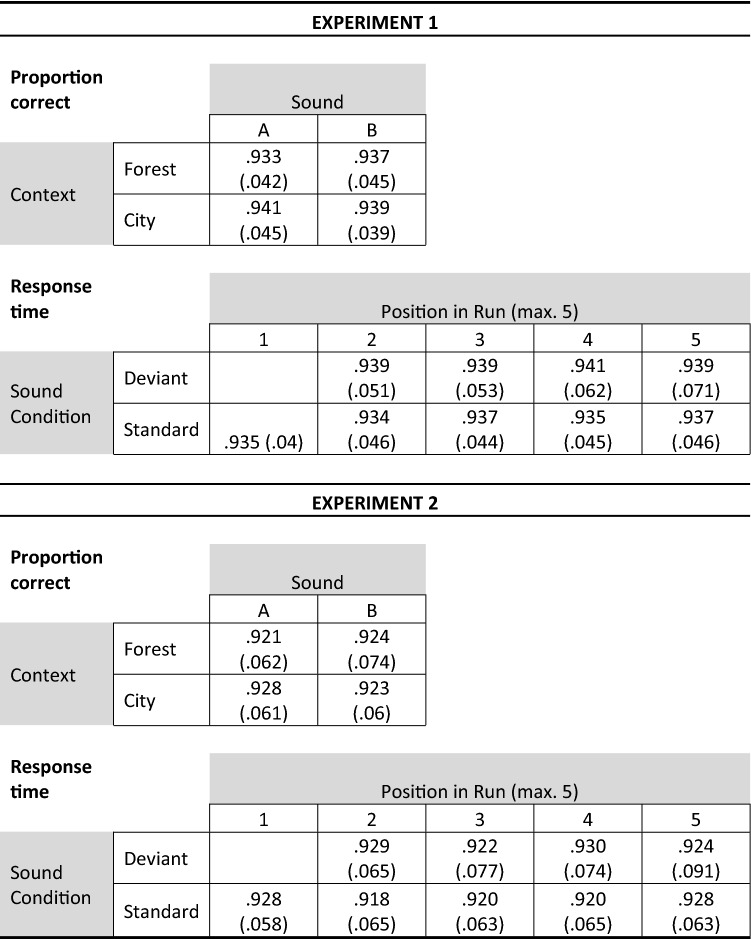
Mean proportions of correct responses in Experiments 1 and 2 as a function of the environmental context (forest, city) and the sound (A, B). Mean proportions of correct responses in Experiment 1 and 2 for context-based standard and deviant sounds as a function of the position of the trial within a run of trials sharing the same context. Numerical values within brackets represent the standard deviation.

**Figure 2:**
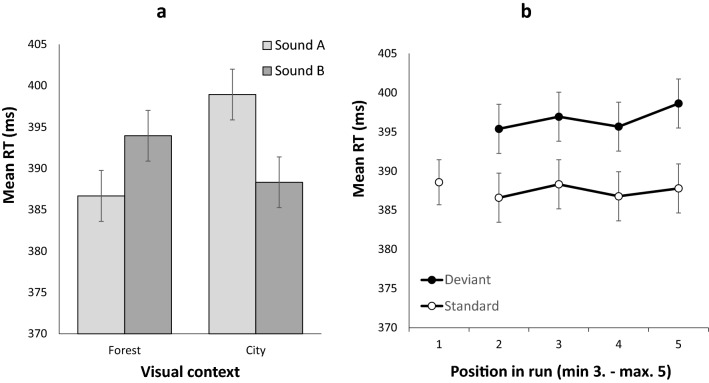
Panel (a): Mean response times in Experiment 1 as a function of the environmental context (forest, city) and the sound (A, B). In the forest context, sounds A and B were presented with probabilities of .882 and .118, respectively. These probabilities were reversed in the city context. Panel (b): Mean response times in Experiment 1 for context-specific standard and deviant sounds as a function of the position of the trial within a run of trials sharing the same context. Error bars represent one standard error of the mean.

A similar analysis was carried out on the mean response time for correct responses. The main effect of Context was significant (faster responses in the forest context, *M* = 390.310 ms, *SD* = 45.559 ms, than in the city context, *M* = 393.623 ms, *SD* = 46.062 ms), *F*(1,55) = 11.825, *MSE* = 51.974, *p* < 0.001, ${\eta }_{p}^{2}$ = 0.177, *BF*_*10*_ = 4.881. The main effect of Sound just reached significance, *F*(1,55) = 4.001, *MSE* = 38.802, *p* = 0.050, ${\eta }_{p}^{2}$ = 0.068, but was not supported by the Bayes Factor (*BF*_*10*_ = 0.342). Most importantly, a significant interaction was observed between these factors, *F*(1,55) = 65.005, *MSE* = 68.928, *p* < 0.001, ${\eta }_{p}^{2}$ = 0.542, *BF*_*10*_ = 5,020,378.710 (see Fig. 2A). Follow-up tests revealed that, within each context, RTs were significantly longer for the least frequent sound relative to the most frequent sound: Sound B relative to Sound A in the forest context (*t*(55) = -5.458, *p* < 0.001, *d* = -0.729 (95%CI: -1.022 to -0.432), BF_*10*_ = 13,641.672), and Sound A relative to Sound B in the city context, (*t*(55) = 7.377, *p* < 0.001, *d* = 0.986 (95%CI: 0.663 to 1.303), BF_*10*_ = 2.374 × 10^13^).

### Temporal dynamics across same context runs

A follow-up analysis was carried out to examine the temporal dynamics of distraction across runs of trials involving the same context (forest or city). Such runs contained 3 to 5 trials and always started with a trial in which the sound presented corresponded to the standard sound within the context in question. The aim of this analysis was to determine how quickly the cognitive system reconfigured its auditory predictions upon a change in environmental context. More specifically, it allowed us to determine how a sound that recently acted as a deviant sound in one context (e.g., sound B in the forest context) was processed when constituting a standard sound in the newly changed context (e.g., sound B in the city context).

The analysis was carried out in two steps. First, we compared the mean performance in the first trial of a run (always standard; STD1) to the second trial (standard or deviant; STD2 or DEV2 respectively). Second, mean performance in trials 2 to 4 of a run were analyzed using a 2 (Sound Condition: standard vs deviant) × 4 (Position: trial 2–5 within a run) ANOVA for repeated measures. These analyses were carried out on both dependent variables (proportion correct and RTs, with the second being of primary interest).

The mean proportion of correct responses (see Table 1) was comparable across all sound conditions and positions. No difference was observed between STD1 and STD2 (*t*(55) = 0.239, *p* = 0.812, *d* = 0.032 (95% CI -0.230 to 0.294), *BF*_*10*_ = 0.150), or between STD1 and DEV2 (*t*(55) = -0.620, *p* = 0.538, *d* = 0.083 (95% CI -0.345to 0.180), *BF*_*10*_ = 0.175). The Sound Condition x Position (2–5) ANOVA revealed no main effect of Sound Condition (*F*(1,55) = 0.899, *MSE* = 0.002, *p* = 0.347, ${\eta }_{p}^{2}$ = 0.016, *BF*_*10*_ = 0.173), no main effect of Position *F*(3,165) = 0.074, *MSE* = 0.001, *p* = 0.974, ${\eta }_{p}^{2}$ = 0.001, *BF*_*10*_ = 0.010), and no interaction (*F*(3,165) = 0.090, *MSE* = 0.001, *p* = 0.965, ${\eta }_{p}^{2}$ = 0.002, *BF*_*10*_ = 0.020).

In contrast, the analysis of RTs revealed more interesting effects. The mean RT on the first trial (STD1) was similar to that on the second standard trial (STD2; *t*(55) = 1.581, *p* = 0.120, *d* = 0.211 (95% CI -0.054 to 0.475), *BF*_*10*_ = 0.470) but was significantly faster than the mean RT on the deviant trial in second position (DEV2; *t*(55) = -3.289, *p* = 0.002, *d* = -0.439 (95% CI -0.712 to -0.163), *BF*_*10*_ = 16.555). Across positions 2 to 5, responses were slower for deviant than for standard trials, *F*(1,55) = 75.056, *MSE* = 128.600, *p* < 0.001, ${\eta }_{p}^{2}$ = 0.577, *BF*_*10*_ = 1.760 × 10^14^. The main effect of Position was not significant, *F*(3,165) = 0.964, *MSE* = 134.000, *p* = 0.411, ${\eta }_{p}^{2}$ = 0.017, *BF*_*10*_ = 0.029, and nor was the interaction between these factors, *F*(3,165) = 0.276, *MSE* = 110.192, *p* = 0.843, ${\eta }_{p}^{2}$ = 0.005, *BF*_*10*_ = 0.029. In summary, a sound acting as a deviant in a run of trials sharing one context ceased to slow responses in the very first trial following a change of context (i.e., when it now constituted a standard sound; see Fig. 2B).

## Discussion

The results of Experiment 1 are unambiguous: The impact of task-irrelevant sounds occurring with equal probabilities across the task was modulated by their relationship with the environmental context (here instrumentalized as a task-irrelevant picture of a forest or of a city scene). Task-irrelevant sounds acquired the status of a deviant or a standard sound dependent upon their predictability given the environmental context, such that one sound became distractive relative to the other within one context (e.g., forest scene) while the reverse relationship was observed in the other context (city scene). This finding indicates that participants process the task-irrelevant sound as part of a more global context, integrating information across sensory modalities, even though the primary task made no demand on the auditory modality. Upon a change of context (e.g., forest scene to a city scene), the cognitive system updated its predictions in a context-dependent manner. This updating was completed within 200 ms from the onset of the picture. Indeed, we observed that a sound acting as a deviant sound and yielding longer RTs within one context did not do so on the very first trial following a change of context.

While the implementation of the environmental context inspired from previous work using visual search tasks^54,55^ proved efficient in our simple left–right categorization task, it is interesting to reflect upon the nature of this environmental context. In Experiment 1, this context consisted of two fixed visual scenes across the task^54^, though in our experiment the selection of the pictures varied across participants, as in previous work^55^. Hence, the sounds may have been associated with any, or several, of many features characterizing the context: its visual features (e.g., colors, shapes, geometrical aspects) or its lexico-semantic features (interpretation of the scene as that of a forest or of that of a city scene, with all its possible ramifications). Our manipulation certainly contributed to establish solid and distinct environmental contexts. Yet, given the results from Experiment 1 and the apparent speed with which the cognitive system updates its sensory predictions, it would be interesting to examine whether the heterogeneity of the environmental context can influence the sound-context association by putting participants to the test using environmental contexts that are perceptually changing but categorically stable. We explored this issue in Experiment 2 by modifying our task to use sets of forest and city scenes and, while maintaining runs of 3 to 5 trials of a same context, varying the background picture on every trial. In doing so, we aimed to establish whether the cognitive system can differentiate between the two contexts in the face of constantly changing perceptual features. If it can, we aimed to examine how quickly the cognitive system updates its sensory predictions after a change of context.

## Experiment 2

### Results

#### Deviance distraction as a function of visual context

The mean proportion of correct responses (see Table 1) was analyzed using a 2 (Context: forest vs city) × 2 (Sound: A vs B) ANOVA for repeated measures. The main effect of Context was not significant, *F*(1,51) = 0.655, *MSE* = 6.882 × 10^–4^, *p* = 0.422, ${\eta }_{p}^{2}$ = 0.013, *BF*_*10*_ = 0.221, nor was that of Sound, *F*(1,51) = 0.052, *MSE* = 5.423 × 10^–4^, *p* = 0.821, ${\eta }_{p}^{2}$ = 0.001, *BF*_*10*_ = 0.152. The interaction between these factors was not significant either, *F*(1,51) = 2.296, *MSE* = 3.621 × 10^–4^, *p* = 0.136, ${\eta }_{p}^{2}$ = 0.043, *BF*_*10*_ = 0.412.

**Figure 3:**
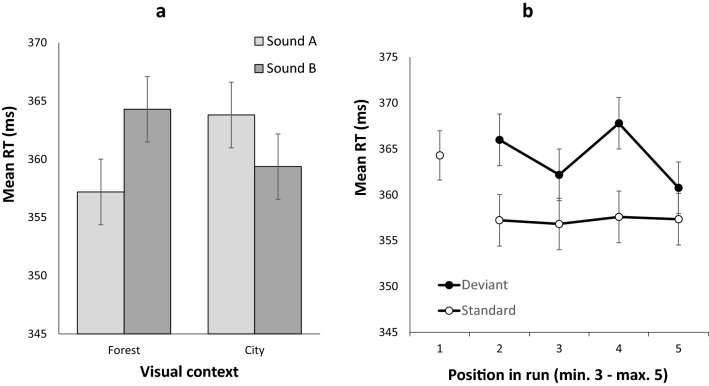
Panel (a): Mean response times in Experiment 2 as a function of the environmental context (forest, city) and the sound (A, B). In the forest context, sounds A and B were presented with probabilities of .882 and .118 respectively. These probabilities were reversed in the city context. Panel (b): Mean response times in Experiment 2 for context-specific standard and deviant sounds as a function of the position of the trial within a run of trials sharing the same context. Error bars represent one standard error of the mean.

The analysis of RTs revealed no main effect of Context (*F*(1,51) = 0.372, *MSE* = 45.887, *p* = 0.372, ${\eta }_{p}^{2}$ = 0.016, *BF*_*10*_ = 0.210) or Sound (*F*(1,51) = 2.841, *MSE* = 32.468, *p* = 0.098, ${\eta }_{p}^{2}$ = 0.053, *BF*_*10*_ = 0.344). However, the Context x Sound interaction was significant: *F*(1,51) = 35.751, *MSE* = 48.351, *p* < 0.001, ${\eta }_{p}^{2}$ = 0.412, *BF*_*10*_ = 4,020,378.710 (see Fig. 3A). Follow-up tests revealed that, within each context, RTs were significantly longer for the least frequent sound relative to the most frequent sound: Sound B relative to Sound A in the forest context (*t*(51) = -7.798, *p* < 0.001, *d* = -0.665 (95%CI: -0.963 to -0.362), BF_*10*_ = 1353.247), and Sound A relative to Sound B in the city context, (*t*(51) = 4.623, *p* < 0.001, *d* = 0.641(95%CI: 0.340 to 0.937), BF_*10*_ = 779.792).

#### Temporal dynamics across same context runs

The mean proportion of correct responses (see Table 1) was slightly higher in STD1 than in STD2 (M = 0.931, SD = 0.056, vs M = 0.920, SD = 0.063, *t*(51) = 2.848, *p* = 0.006, *d* = 0.395 (95% CI 0.111 to 0.675), *BF*_*10*_ = 5.499), but did not differ for STD1 and DEV2 (*t*(51) = -0.215, *p* = 0.831, *d* = -0.030 (95% CI -0.302 to 0.242), *BF*_*10*_ = 0.154). The Sound Condition x Position (2–5) ANOVA on the proportion of correct responses revealed no main effect of Sound Condition (*F*(1,51) = 2.868, *MSE* = 8.109 × 10^−4^, *p* = 0.096, ${\eta }_{p}^{2}$ = 0.053, *BF*_*10*_ = 0.276), no main effect of Position *F*(3,153) = 0.331, *MSE* = 0.002, *p* = 0.803, ${\eta }_{p}^{2}$ = 0.006, *BF*_*10*_ = 0.017), and no interaction (*F*(3,153) = 1.053, *MSE* = 0.001, *p* = 0.371, ${\eta }_{p}^{2}$ = 0.020, *BF*_*10*_ = 0.083).

The mean RT on the first trial (STD1) was significantly slower compared to that on the second standard trial (STD2; *t*(51) = 7.257, *p* < 0.001, *d* = 1.006 (95% CI 0.669 to 1.337), *BF*_*10*_ = 5.41 × 10^6^) and was comparable to the RT on DEV2 (*t*(51) = -1.138, *p* = 0.260, *d* = -0.158 (95% CI -431 to 0.116), *BF*_*10*_ = 0.278). Across positions 2 to 5, responses were slower for deviant than for standard trials: *F*(1,51) = 33.888, *MSE* = 147.954, *p* < 0.001, ${\eta }_{p}^{2}$ = 0.399, *BF*_*10*_ = 3.835 × 10^7^. Some variation was observed across positions but was not supported by Bayesian statistics (*F*(3,153) = 2.775, *MSE* = 111.691, *p* = 0.043, ${\eta }_{p}^{2}$ = 0.052, *BF*_*10*_ = 0.240). No interaction was observed between these factors: *F*(3,153) = 2.376, *MSE* = 105.286, *p* = 0.072, ${\eta }_{p}^{2}$ = 0.045, *BF*_*10*_ = 0.357. These results are illustrated in Fig. 3B. In summary, a sound acting as a deviant sound and slowing responses within the previous context, remained distracting on its first occurrence as a standard sound within the new context. However, from the second trial in each run, the standard and deviant sounds for the new context diverged in their response times, consistent with the influence of the changed context.

#### Comparing fixed versus varying contexts

Experiment 2 differed from Experiment 1 in that it involved many pictures in each of the two environmental contexts (forest and city). To assess whether the two types of manipulation differed with respect to the processing of the environmental context information, we compared the deviance distraction effect between the two experiments. To do so, we computed the mean distraction observed in each context (forest: RT_SoundB_—RT_SoundA_; city: RT_SoundA_—RT_SoundB_) and tested the hypothesis that context-specific distraction by the unexpected sound should be greater when this context is defined by a single picture as opposed to several pictures (one-tailed t-test). The context-dependent distraction effect was significantly larger for the fixed context (*M* = 8.945, *SD* = 8.302) than for the varying context (*M* = 5.766, *SD* = 6.953), *t*(106) = 2.149, *p* = 0.017, *d* = 0.414 (95%CI 0.093 to infinity), BF_10_ = 3.077.

## Discussion

The results of Experiment 2 were clear-cut: The impact of the task-irrelevant sounds was again modulated by the environmental context. Slower responses were observed in the visual categorization task in trials involving the sound that was least expected within a specific context. Put differently, the same sound produced shorter response times within the context in which it was predictable relative to that in which it was not. Remarkably, this was observed despite a change of background picture on every trial. This may indicate that participants extracted semantic information from each picture, or that they extracted common information from multiple pictures of the same type of context. Whichever is the case, the data demonstrate that the contextual modulation of deviance distraction is not limited to the associative learning of sounds and specific pictures. In contrast to Experiment 1, the analysis of the temporal dynamics of this effect in Experiment 2 revealed that the reconfiguration of sensory predictions was slower than when contexts were defined by single pictures. Indeed, in Experiment 2, response times in the first standard trial following a change of context (e.g., from forest to city) were slower than in other standard trials and equivalent to those observed for deviant trials within the same context. In other words, a sound that constituted a deviant sound in a recent context (e.g., forest) still exerted distraction when first presented as the standard sound in the other context (e.g., city), and not thereafter.

### General discussion

In this study, we sought to examine whether task-irrelevant sounds are processed in relation to contextual elements of the task. More specifically, we investigated the extent to which sounds that are equally probable across the task would yield distraction when they constitute deviant sounds within a given environmental context (instrumentalized as a picture displayed as a task-irrelevant background in the primary visual task). If auditory predictions were generated on the basis of modality-specific information and were, therefore, independent from the visual context, performance in the categorization task should have remained stable irrespective of combination of sound and environmental context. Our results clearly departed from this prediction and, instead, support the hypothesis that sounds, although equiprobable across the task, do act as deviant and standard sounds depending on their conditional probabilities within a given environmental context. In Experiment 1, our environmental contexts consisted of two pictures: one of a forest and one of a city. The results confirmed that sensory predictions are generated in a context-dependent manner, such that the sound rarely presented within one specific context yielded longer response times in the primary task relative to the sound frequently associated with that same context. The analysis of this effect across runs of trials of the same context revealed that the cognitive system was capable of reconfiguring its sensory predictions on the very first trial following a change of environmental context. More specifically, this reconfiguration was completed within the 200 ms window during which the visual background was visible ahead of the presentation of the sound. In Experiment 2, the forest and city contexts were implemented using a large set of pictures that changed on every trial. Despite the constant change of background picture, the main result was identical to that of Experiment 1: Longer RTs were observed in the primary task in the presence of a task-irrelevant sound when this sound’s probability of occurrence was low in a given context (forest vs city) relative to a sound that was highly probable within that context. However, a subsequent analysis revealed that, in contrast to Experiment 1, participants took relatively longer to reconfigure their sensory predictions, such that the sound most likely to occur within a given context did produce distraction on the very first trial following a change of context. In other words, when the environmental context is defined categorically rather than by a specific picture, the context-dependent reconfiguration of predictions appears to require more time.

Evidence from object recognition and detection studies using visual scenes as environmental context indicates that semantic aspects of these scenes are processed automatically and rapidly, including under subliminal presentation times^69–73^. Participants are certainly capable of understanding a visual scene with exposure durations of about 100 ms^69,74,75^ and to extract contextual semantic information in as little as 80 ms^76^. In our experiments, while our visual scenes were more complex that single objects, their appearance 200 ms ahead of the sound’s onset should have allowed participants sufficient time to process the contextual information and distinguish one context from the other. Yet our results suggest that the environmental context and its statistical relation to the task-irrelevant sound may involve different mechanisms when context is defined by a single picture (Experiment 1) compared to varying pictures (Experiment 2). Indeed, in Experiment 2 we observed less deviance distraction and a slower updating of sensory predictions. A context defined by a fixed picture affords constant perceptual (shapes, configuration of elements, colors, etc.) and semantic features, thereby maximizing the amount of information available to the cognitive system to identity and learn that context. One possibility is that the cognitive system combines all of these features in the representation of the context in relation to the task-irrelevant sounds. However, this does not necessarily need to be the case, for one may argue that a fast and efficient way to process constant contextual information would be to limit it to low-level visual features (since these are presumably processed fastest). When the context consists of a fixed picture, such information is sufficient to distinguish it from an alternative context. What is clear, however, is that in the case of contexts defined by varying pictures, context-dependent effects must necessarily involve the extraction of features common to these pictures. Such features may be perceptual, for these pictures would, for example, share certain color schemes (forests tend to be characterized by green, brown, yellow and red tones, while city scenes are more likely to contain other tones, such as grays or blues) or geometrical arrangements (forests contain several vertically oriented elements such as tree trunks and are dominated by texture zones and undulating contours, while city scenes tend to be characterized by straight lines^77,78^). The information extracted may also be categorical (lexico-semantic), freeing the context from its sensory features and rendering it more abstract. Whichever type of information is extracted, one advantage of such abstraction would be to process environmental contexts in a more flexible manner, allowing for changes in the scenes while preserving the context’s coherence. It is interesting to note that research on a different type of visual stimuli, namely human faces, has shown that participants seem to encode the average of such stimuli with respect to aspects such as emotional expression^79^, gender^80^ or gaze direction^81^. Such extraction of summary statistics has been observed with both familiar^82^ and unfamiliar^83^ faces. One aspect worth considering is the distinction between the extraction of information from a visual context and the categorization of this context. While evidence suggests that participants presented with very brief (94 ms) black and white pictures depicting natural (e.g., forest, river) or man-made scenes (e.g., house, construction) are capable of categorizing these correctly^84^, such a task differs from ours in that it explicitly requires participants to attend to and categorize visual scenes. In our experiment, the environmental context was incidental to the task and participants were encouraged to ignore it to focus on the target stimulus. Interestingly, evidence based on multivariate pattern analysis of EEG signals indicates that the incidental categorization of color scenes such as roads, churches, houses and supermarkets, appears to take about 200 ms^85^. This suggests that in our Experiment 2, the categorization of our forest or city scenes would barely have been completed (or indeed on many occasions it may not have) by the time the task-irrelevant sound was presented. Under the reasonable assumption that the reconfiguration of sensory predictions would follow the categorization of the context (be it in perceptual and/or semantic terms, our position is agnostic in this respect), the sound would be processed ahead of this reconfiguration being completed. Hence, the environmental context would fail to trigger the reconfiguration of the predictions in time to complete the very first trial following a change of context. However, such reconfiguration would be completed by the next trial. In contrast, in Experiment 1, the reconfiguration could be achieved significantly faster because the use of a fixed picture per context rendered unnecessary the categorization of the context based on some abstracted information.

Our results provide further evidence that the environmental context is processed even when it is task-irrelevant and when the primary task makes no demand on explicit memory. Indeed, in contrast to past studies reporting the impact of the context of recall on recollection performance^44–46,50^, participants in our study simply categorized visual stimuli without any requirement to voluntarily encode any information for later recall. This suggests that the associative learning of task-relevant and task-irrelevant information and predictions generated upon it are not process-dependent. That is, the cognitive system need not be engaged in explicit memory processes for this learning to occur. In that respect, our results add to the limited evidence on the impact of the context on attentional tasks. Indeed, past work manipulating the environmental context in visual attention tasks^54,55^ reported clear indications that visual search mechanisms are modulated by the reinstatement of information previously associated with specific contexts. In contrast to these studies, however, ours extended this demonstration to a cross-modal task. To our knowledge, our study constitutes the first demonstration of the cognitive system’s associative learning of irrelevant visual information (environmental context) and irrelevant auditory information in the context of a visual primary task. This suggests that task elements, though irrelevant, are processed and bound at a level of cognitive hierarchy that supersedes sensory or modality-specific codes. Remarkably, this associative learning can involve a relatively complex abstraction of the context, as was the case when it is not defined by a single visual scene but by several, reinforcing the contention that this associative learning cannot be reduced to the binding of sensory information. When the context is abstracted, the cognitive system takes longer to adapt its predictions. This may reflect the time required to activate the abstracted context from a specific visual scene, and/or the longer time required to update auditory predictions when the impetus to do so emanates from higher levels in the cognitive architecture, referred to as “episodic control” by Koechlin et al.^63^, rather than from sensory control. Our study certainly opens avenues for future research, from a parametric exploration of the temporal dynamics of the context-dependent reconfiguration of sensory predictions (e.g., how long does the cognitive system take to update predictions?), to the further study of the mental representation of the environmental context (e.g., what is the nature of context abstraction when defined by multiple images?).

Finally, it is worth considering briefly some potential broader implications of our findings. We showed that task-irrelevant sounds are processed in relation to the environmental context, which we instrumentalized as a task-irrelevant background picture. However, environmental contexts arguably involve more than the screen background (e.g., the room, building or wider geographical context, or other circumstantial or episodic elements, such as the experience of participating in an experiment). Hence, our environmental context manipulation was only partial. This means that the actual impact of the environmental context on deviance distraction may potentially be stronger than the effect we measured in our experiments. By the same token, in past studies where the environmental context was not experimentally manipulated and all auditory stimuli were processed within the same broad context (e.g., constant screen background, room, etc.), it is possible that the effect of deviant sound (electrophysiological and/or behavioral) reported in these studies may have, at least partially, emerged through context-dependent mechanisms. Unsurprisingly, past studies have not considered this aspect. Yet, it presents certain implications, for it suggests that sensory predictions may not be solely underpinned by the activity of sensory, posterior, brain regions but may also involve links to the activity of other sensory regions (e.g., visual) and/or higher (frontal) regions. Furthermore, our results may also possibly lead to the reinterpretation of some past findings. For example, several studies have concluded that the reduction of deviance distraction when standard and deviant sounds are preceded by visual cues reflect the intervention of cognitive control^34,35,86^. However, if the visual cue is interpreted as part of the environmental context, then this finding can be explained as a manifestation of the context-dependent processing of the auditory stimuli without the need to invoke cognitive control (the underpinning mechanisms of which past studies have not clearly delineated).

In summary, our study shows that participants performing a visual categorization task involuntarily process task-irrelevant stimuli and their relationship to the environmental context. As a result, the presentation of the latter appears to cue the updating of sensory predictions, thereby resulting in distraction when a given sound deviates from predictions. Our results indicate that task-irrelevant sounds are not only processed in relation to the auditory context (built over time and defined as past auditory stimuli^32,35,87–89^) but also, across sensory modalities, and rapidly, to the visual environmental context.

## Methods

### Experiment 1

#### Participants

Fifty-six participants aged 19–42 (M = 20.714, SD = 5.150) took part in this study (46 females). Four participants were lefthanded. All were undergraduate psychology students who took part in the study in exchange for course credit.

#### Material and stimuli

A set of five forest scenes and five city scenes was built. These pictures were selected from a royalty-free photography repository (pixabay.com). Pictures were resized or cropped to a 1280 × 720 pixels dimension. None of the pictures contained prominent people, vehicles, or readable text.

The task was programmed using Psychology Software’s E-Prime 3.0 software and was executed on a PC computer equipped with a 17in screen. Auditory stimuli were delivered binaurally with headphones, at an intensity of approximately 70 dB SPL. Participants were tested within a sound-attenuated cabin.

#### Procedure

Participants were asked to categorize the direction in which a target stimulus (“ < “ or “ > “) pointed, while ignoring task-irrelevant sounds presented immediately before each target stimulus as well as pictures displayed in the background.

Each trial began with the appearance of a background picture occupying the entire screen (referred to as context hereafter), and a fixation cross in white color in the middle of a centrally located black rectangle that occupied 13% of the screen’s width and 18% of the screen’s height. These stimuli remained visible throughout the trial, except when the target stimulus temporarily replaced the fixation cross (as described below). Following an interval of 200 ms, one of two task-irrelevant sinewave sounds (440 Hz or 1047 Hz) was presented for 200 ms. Upon the sound’s offset, the target stimulus (“ < “ or “ > “, in Arial font size 48) replaced the fixation cross for a duration of 200 ms, followed by a further interval of 800 ms before the next trial began automatically. A schematic illustration is presented in Fig. 1.

The choice and arrangement of the background pictures, sounds and target stimuli followed specific rules. Across the task, one forest picture and one city picture were used. For each participant, these pictures were selected randomly from respective sets of five pictures. The two selected pictures were used equiprobably as background pictures in runs of trials of 3 to 5 consecutive trials, creating a total of 1216 test trials. That is, there were 3–5 trials with one background picture, then 3–5 trials with the other background picture without any change in the continuity of trials, and so on in alternation. Among these forest and city trials, both target stimuli (“ < “ and “ > “) were presented equally often. Two irrelevant sounds (440 Hz and 1047 Hz) were used equiprobably across the task but with distinct probabilities within each of the two contexts (forest vs city). One sound (e.g., 440 Hz) was more likely in one context (e.g., forest context) than the other (e.g., 1047 Hz): *p* = 0.882 and *p* = 0.118, respectively. These probabilities were reversed in the other context (e.g., city). Hence, while each sound was presented in half the trials across the task, it constituted a standard sound in one context (e.g., forest) and a deviant sound in the other (e.g., city). The allocation of the sounds (A and B) to the two contexts (forest and city) was counterbalanced across participants. Within a run of trials of the same context, sounds constituting a deviant within that context were never presented on the first trial of a run. Finally, only half of the runs of trials of a specific context involved a trial with a deviant sound (to avoid expectation effects). The 1216 test trials were divided into 4 blocks of 304 trials lasting about 7 min each (participants were allowed to pause for a few seconds before resuming the task).

Before participants completed the 4 blocks of test trials, they performed a block of 10 practice trials in which no sound or background picture was presented. Feedback was provided after each response in the form of text appearing during 1500 ms after the participant’s response. This feedback indicated whether the response was correct, incorrect, or urged participants to respond faster if they failed to respond. This feedback was not presented in the rest of the experiment.

### Experiment 2

#### Participants

Fifty-eight psychology undergraduate students took part in this online study in exchange for course credit (none had taken part in Experiment 1). Six reported having been interrupted during the experiment and were removed from the sample, leaving 52 participants (42 females) aged 19 to 26 (M = 19.692, SD = 1.365). Six participants were lefthanded.

#### Material and stimuli

A set of 32 forest pictures and 32 street pictures was built. These pictures were selected from four royalty-free photography repositories (unplash.com, pixabay.com, freeimages.com, pixels.com). Where necessary, pictures were resized or cropped to a 1280 × 720 pixels dimension. None of the pictures contained people, vehicles, or readable text (in a few cases, this was achieved though airbrushing out some information).

Since this experiment was carried out online, the exact characteristics of the computers used to run the experiment, such as monitor size, is not known. The task was programmed using Psychology Software’s E-Prime 3.0 software and deployed using EPrimeGo 1.0 (running on PC computers only). Participants were instructed to wear headphones or, alternatively, use loudspeakers connected to their computer.

#### Procedure

The cross-modal oddball task followed the same design as in Experiment 1 with the following differences. Across the task, 32 forest pictures and 32 city pictures were used, with each picture used 19 times across the experiment. The order of presentation of these pictures was set so that each set of 32 pictures was used once (in random order) before a new random cycle was presented. The same picture was never presented on successive trials.

Since the task was administered online, it was not possible to control sound levels or equate them across participants. An audio–video introduction was presented at the beginning of the experiment inviting participants to wear headphones (or use loudspeakers) and set the sound level on their computer to a comfortable level. Sound checks were programmed within the task at the onset of the experiment and after the last trial of every block. These tests consisted in the presentation of a sequence of 3 auditory digits that participants were required to reproduce in order using the corresponding keys on their keyboards. Participants were told that these checks would take place at different moments during the experiments. These checks were implemented to reduce the risk that participants would turn the sound off.

Finally, at the end of the experiment, participants were asked to indicate whether they were interrupted while performing the experiment (data from participants who declared having been interrupted during the experiment were discarded from the analysis).

This study was carried out in accordance with the recommendations of American Psychological Association with written informed consent from all subjects. All subjects gave written informed consent in accordance with the Declaration of Helsinki. The protocol was approved by the Bioethical Committee of the University of the Balearic Islands.

## Data Availability

The dataset of this study (Experiments 1 and 2) is available on the Open Science Framework: https://osf.io/6fhwq/.
